# The Evolution of the Epidemic of Charcoal-Burning Suicide in Taiwan: A Spatial and Temporal Analysis

**DOI:** 10.1371/journal.pmed.1000212

**Published:** 2010-01-05

**Authors:** Shu-Sen Chang, David Gunnell, Benedict W. Wheeler, Paul Yip, Jonathan A. C. Sterne

**Affiliations:** 1 Department of Social Medicine, University of Bristol, Bristol, United Kingdom; 2 Ju Shan Hospital, Taoyuan, Taiwan; 3 Department of Social Work and Social Administration, the University of Hong Kong, Hong Kong SAR, China; 4 The Centre for Suicide Research and Prevention, the University of Hong Kong, Hong Kong SAR, China; University of Western Sydney, Australia

## Abstract

**Background:**

An epidemic of carbon monoxide poisoning suicide by burning barbecue charcoal has occurred in East Asia in the last decade. We investigated the spatial and temporal evolution of the epidemic to assess its impact on the epidemiology of suicide in Taiwan.

**Methods and Findings:**

Age-standardised rates of suicide and undetermined death by charcoal burning were mapped across townships (median population aged 15 y or over = 27,000) in Taiwan for the periods 1999–2001, 2002–2004, and 2005–2007. Smoothed standardised mortality ratios of charcoal-burning and non-charcoal-burning suicide and undetermined death across townships were estimated using Bayesian hierarchical models. Trends in overall and method-specific rates were compared between urban and rural areas for the period 1991–2007. The epidemic of charcoal-burning suicide in Taiwan emerged more prominently in urban than rural areas, without a single point of origin, and rates of charcoal-burning suicide remained highest in the metropolitan regions throughout the epidemic. The rural excess in overall suicide rates prior to 1998 diminished as rates of charcoal-burning suicide increased to a greater extent in urban than rural areas.

**Conclusions:**

The charcoal-burning epidemic has altered the geography of suicide in Taiwan. The observed pattern and its changes in the past decade suggest that widespread media coverage of this suicide method and easy access to barbecue charcoal may have contributed to the epidemic. Prevention strategies targeted at these factors, such as introducing and enforcing guidelines on media reporting and restricting access to charcoal, may help tackle the increase of charcoal-burning suicides.

*Please see later in the article for the Editors' Summary*

## Abstract 2

Shu-Sen Chang and colleagues describe the epidemiology of an epidemic of suicide by charcoal burning in Taiwan and discuss possible reasons for its spread.

## Editors' Summary

**Background:**

Every year, about a million people take their own lives. Most people who die by suicide are mentally ill but some people take their lives because stressful events (the loss of a partner, for example) have made life seem worthless or too painful to bear. Strategies to reduce suicide rates include better treatment of mental illness and programs that help people at high risk of suicide deal with stress. Suicide rates can also be reduced by limiting access to common suicide methods. These methods differ from place to place. Hanging is the predominant suicide method in many countries but, in Hong Kong, for example, jumping from a high building is the commonest method. Suicide methods also vary over time. In 1998, a woman in Hong Kong took her life by burning barbecue charcoal in a sealed room (a process that produces high levels of the toxic gas carbon monoxide). This method was unheard of before and was extensively reported by the mass media; by the end of 2004, charcoal-burning suicide became the second most common form of suicide in Hong Kong.

**Why Was This Study Done?:**

The epidemic of charcoal-burning suicide that started in Hong Kong has rapidly spread to other countries in East Asia, including Taiwan, where it is also now the second most common method of suicide. It would be useful to identify the factors that have contributed to the spread of this particular form of suicide because such knowledge might help to improve strategies for preventing charcoal-burning suicide. One way to identify these factors is to examine the space–time clustering of charcoal-burning suicides. Clustering of specific types of suicides in both time and space usually occurs in settings such as institutions where the individuals who die by suicide have been in social contact. By contrast, clustering of specific types of suicide in time more than place is often associated with media coverage of events such as celebrity suicides, which can lead to imitative suicides. In this study, therefore, the researchers investigate the evolution of the epidemic of charcoal-burning suicide over time and across areas in Taiwan.

**What Did the Researchers Do and Find?:**

The researchers obtained data on suicides and undetermined deaths (most “missed” suicides are recorded as undetermined deaths) from 1999 to 2007 from the Taiwan Department of Health. They then used statistical methods to estimate the standardized mortality rates (the ratio of the observed to the expected numbers of deaths) of charcoal-burning and non-charcoal-burning suicides and undetermined deaths in different areas of Taiwan. The proportion of suicides that were charcoal-burning suicides rose from 0.1% in 1991 to 26.6% in 2007, they report, and the epidemic of charcoal-burning suicide was more marked in urban than in rural areas. However, there was no single point of origin of the epidemic. Finally, they report, rates of charcoal-burning suicide were consistently higher in urban than in rural areas throughout the study period, a result that means that, although overall suicide rates were higher in rural than in urban regions of Taiwan prior to the epidemic of charcoal-burning suicide, the difference has now almost disappeared.

**What Do These Findings Mean?:**

These findings suggest that the epidemic of charcoal-burning suicide may underlie recent changes in the geography of suicide in Taiwan. However, the study's findings may not be numerically accurate because of some of the assumptions made by the researchers. For example, there is no specific code for charcoal-burning suicides in official records so the researchers assumed that suicides classified as “poisoning using nondomestic gas” were all charcoal-burning suicides, although other studies have shown that nearly 90% of deaths in the category were indeed charcoal-burning suicides. Nevertheless, the observed geographical pattern of charcoal-burning suicides and the changes in this pattern over time suggest that widespread media coverage and easy access to barbecue coal in supermarkets and convenience stores may have contributed to the epidemic of charcoal-burning suicide and to the increase in overall suicide rate in Taiwan and elsewhere in East Asia. Thus, guidelines that encourage responsible media reporting of charcoal-burning suicide (that is, reporting that does not contain detailed descriptions of the method or suggest that this type of suicide is easy and painless) and strategies that restrict access to barbecue charcoal may help to halt the epidemic of charcoal-burning suicide in East Asia.

**Additional Information:**

Please access these Web sites via the online version of this summary at http://dx.doi.org/10.1371/journal.pmed.1000212.

- Another *PLoS Medicine* research article by David Studdert and colleagues investigates the relationship between changes in vehicle emissions laws and the incidence of suicide by motor vehicle exhaust gas in Australia
- The World Health Organization provides information on the global burden of suicide and on suicide prevention (in several languages); see also the article Methods of Suicide: International Suicide Patterns Derived from the WHO Mortality Database
- The US National Institute of Mental Health provides information on suicide and suicide prevention
- The UK National Health Service Choices website has detailed information about suicide and its prevention
- MedlinePlus provides links to further resources about suicide (in English and Spanish)
- The Taiwan Suicide Prevention Center provides information on suicide and its prevention in Taiwan (in Chinese)
- The Centre for Suicide Research and Prevention, the University of Hong Kong, provides information on suicide and its prevention in Hong Kong (in Chinese and English)

## Introduction

The last decade has witnessed an epidemic of suicides by burning charcoal in some Asian countries [1]. The first widely publicised victim died in Hong Kong in November 1998 [1],[2]. She burnt barbecue charcoal in a sealed room to produce high levels of lethal carbon monoxide. Within 5 y this novel method had become the second most commonly used method of suicide in Hong Kong and Taiwan and may have contributed to the rise in overall suicide rates seen in these countries [3],[4]. The rise in use of this method was thought to be triggered by extensive media coverage, which vividly portrayed charcoal burning as an effective, painless, and peaceful way of ending one's life [1],[3],[5]. The new suicide method spread beyond country boundaries [1],[3]; cases have been reported in other parts of Asia including Macau, Japan [1], and Korea [6], and elsewhere in the world [7]. The geographic pattern of the epidemic has not been systematically investigated, although there appears to be an urban–rural difference in incidence in Taiwan [3],[8]. To the best of our knowledge no previous studies have investigated the spatial and temporal pattern of the emergence of a new suicide method.

Different forms of space-time clustering of suicide have been identified in the literature [9]. “Local” clustering refers to occurrences of suicides that group in both space and time: this has been observed mainly in institutional settings such as hospitals, school, or prisons, where victims may be in social contact with each other. Clustering in time more than place is usually associated with media coverage of events such as celebrity suicides that lead to “imitative” suicides [9]. In the case of the charcoal-burning suicide epidemic, where the media influence is thought to be widespread [1],[3], one might predict that there would be little geographic clustering. Alternatively, it is possible that media exposure varies regionally and people living in different areas differ in their susceptibility to media influence; such differences may lead to variation in rates between areas [10]. An improved understanding of the spatial and temporal patterning of charcoal-burning suicide may help identify factors contributing to the epidemic, and thus inform prevention.

This study aimed to investigate the evolution of the epidemic of charcoal-burning suicide over time and across areas in Taiwan, a country with a population of 23 million and large geographic variation in urbanisation. We compared the spatial patterning of charcoal-burning suicides with that of suicides using other methods, and examined the impact of charcoal-burning suicides on secular trends in rural–urban difference in suicide.

## Methods

### Data

Electronic mortality data files for suicides and undetermined deaths (International Classification of Diseases [ICD-9] codes E950–E959 and E980–E989) in the period 1991–2007 were provided by the Department of Health, Taiwan. Past research shows that suicide mortality is often underestimated and the most commonly assigned cause of death for “missed” suicides is death of undetermined intent (“undetermined death”) [11],[12]. A previous study in Taiwan showed that, in addition to undetermined deaths, deaths certified as accident by pesticide poisoning (ICD-9 code E863) and suffocation (ICD-9 code E913) might also contain “missed” suicides [13], and thus we included these deaths in sensitivity analyses. There is no specific code for charcoal-burning suicide in ICD-9 and therefore cases were identified using the codes E952/E982 (suicide or undetermined death by poisoning using nondomestic gas). A previous study showed that up to 90% of deaths in Taiwan in these categories were charcoal-burning cases: the rest were mostly suicides by car exhaust poisoning [14]. For simplicity of presentation, we referred to deaths coded as E952/E982 as “charcoal-burning suicides” throughout the paper. Suicides and undetermined deaths coded other than E952/E982 were analysed as “non-charcoal-burning” cases.

**Figure 1:**
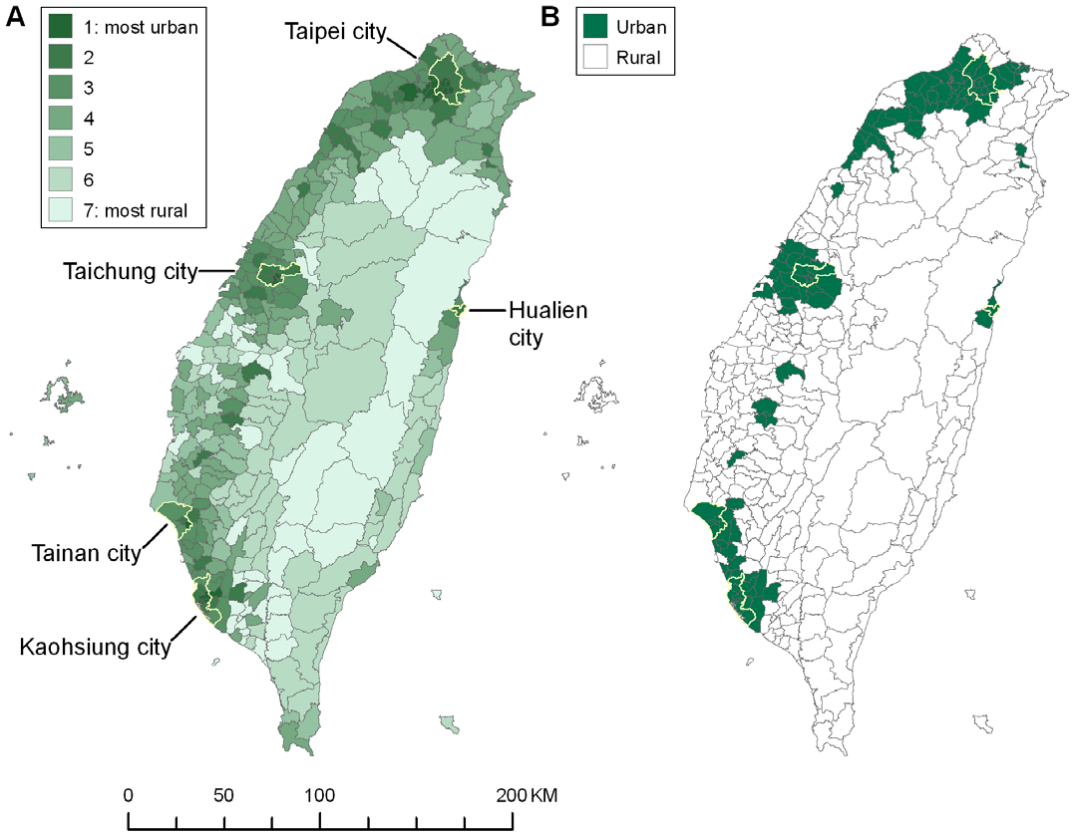
Maps of Taiwanese townships by urbanisation level, 2000. (A) urbanisation levels 1–7; (B) urban (levels 1–3) versus rural (levels 4–7). Five cities are highlighted, including four cities located in the centres of three major metropolitan areas in the north (the capital Taipei city), middle (Taichung city), and south (Tainan and Kaohsiung cities) and one major city in the east (Hualien city).

Mid-year population data for each of the 358 Taiwanese townships (median population aged 15 y or over: approximately 27,000) in each year were obtained from the Demographic Yearbook published by the Ministry of the Interior, Taiwan. Deaths were assigned to each township according to the registered address of residence on the death certificate. We categorised each township as rural or urban on the basis of the urbanisation index developed by the National Health Research Institute, Taiwan [15]. This index was derived from a cluster analysis using data for the year 2000 on five indicators: population density, percentage of population with college or greater educational levels, percentage of population aged 65 y or over, percentage of population working in agriculture, and density of physicians per 100,000 people. Townships are grouped into seven urbanisation levels, with level 1 as the most urban and level 7 as the least (Figure 1A). We categorised townships in levels 1–3 (126 townships, 73% of the population) as urban and those in levels 4–7 (232 townships, 27% of the population) as rural (Figure 1B), in accordance with official statistics that 77% of the residents lived in urban areas in 2000 [16]. Most of the western, plain areas of Taiwan are categorised as urban, with three major metropolitan regions: one in the north (centred around the capital Taipei city), one in the middle (around Taichung city), and one in the south of Taiwan (around Tainan and Kaohsiung cities). Eastern Taiwan is mostly rural and mountainous, with one major city (Hualien city).

### Data Analysis

To investigate changes in spatial patterning over time, sex-specific age-standardised rates of suicide and undetermined death by charcoal burning for each township in the periods 1999–2001, 2002–2004, and 2005–2007 were calculated using the world standard population and mapped. Overall and charcoal-burning–specific rates of suicide as well as the proportions of charcoal-burning suicide were also calculated for each category of urbanisation in men and women during the three time periods. Moran's *I* statistic was used to test for spatial clustering/autocorrelation. *I* = 0 indicates no autocorrelation, whilst *I* = 1 suggests strong spatial autocorrelation (i.e., areas with high rates tend to be proximal to other areas with high rates, and areas with low rates to other areas with low rates). The package GeoDa was used to calculate *I* statistics, allowing for instability of rates due to small numbers of deaths [17],[18]. The *p*-values for *I* statistics were derived from a permutation test.

We calculated standardised mortality ratios (SMRs: the ratio of the observed to expected number of deaths) for each township using data for 1999–2007. Expected deaths were calculated by multiplying the national sex- and age-specific suicide rates (in 5-y age-bands) by the corresponding sex- and age-specific population-years at risk in each township. In areas with small populations the small number of deaths can result in unreliable rate estimates. Therefore Bayesian hierarchical Poisson regression models were used to estimate smoothed SMRs for each township, allowing for “global” between-area variability and “local” variability owing to spatial autocorrelation (i.e., neighbouring areas tend to have more similar rates) [19]–[21]. In the study, “neighbours” were defined as the sets of townships that share a border. In models specifying only global variability, the “globally” smoothed SMR is estimated as a weighted average of the observed value and the national mean: the greater the uncertainty in estimating the area SMRs (in sparsely populated regions), the greater the degree of smoothing towards the national mean. In models specifying only local variability, the “locally” smoothed SMR is a weighted average of the observed SMR and the SMRs in neighbouring areas; the greater unreliability in estimating the SMRs, the higher degree of smoothing towards the local mean. Models specifying both global and local variability (convolution models) allow estimation of the relative contribution of each component to the total variability [22].

Smoothed SMRs were estimated using the Markov chain Monte Carlo methods [23] implemented in WinBUGS version 1.4. The built-in conditional autoregressive distribution was used to smooth SMRs towards the local mean [24]. Vague prior distributions were used. Convergence was assessed using the Gelman-Rubin statistic [25], on the basis of four parallel chains, and models were compared on the basis of the deviance information criterion (DIC).

### Mapping

Smoothed SMRs for suicide and undetermined death by charcoal burning and by other methods were mapped separately, using five category breaks and a divergent red–blue colour scheme [26]. The chosen categories were symmetrical on the logarithmic scale around the middle category (i.e., SMRs of 0.9–1.1) and presented in red with varying lightness for those higher than the middle category, blue for those lower than the middle category, and white for those that belong to the middle category. Five categories were used because past research suggests that people are able to interpret small maps (sized one-quarter of a standard page) better when five shades of colour are used [26]. ArcGIS Version 9.3 was used to produce the maps. In a sensitivity analysis, we analysed data for people aged 25–44 y only, as this age group has a higher risk of charcoal-burning suicide than others [3],[8],[27].

Secular trends in overall and method-specific (charcoal-burning versus non-charcoal-burning) rates for suicide and undetermined deaths for the period 1991–2007 were examined graphically and compared between rural and urban areas in Taiwan.

## Results

Of 53,440 suicides and undetermined deaths in the period 1991–2007, 6,911 (12.9%) were by charcoal burning or other nondomestic gas. The proportion increased from 0.1% in 1991 to 26.6% in 2007. The first charcoal-burning case occurred in Hong Kong in November 1998; during the period 1999–2007, 6,822 (18.7%) of 36,480 suicides and undetermined deaths were by charcoal burning or other nondomestic gas in Taiwan.

### The Emergence of the Epidemic of Charcoal-Burning Suicide over Time and across Areas

**Figure 2:**
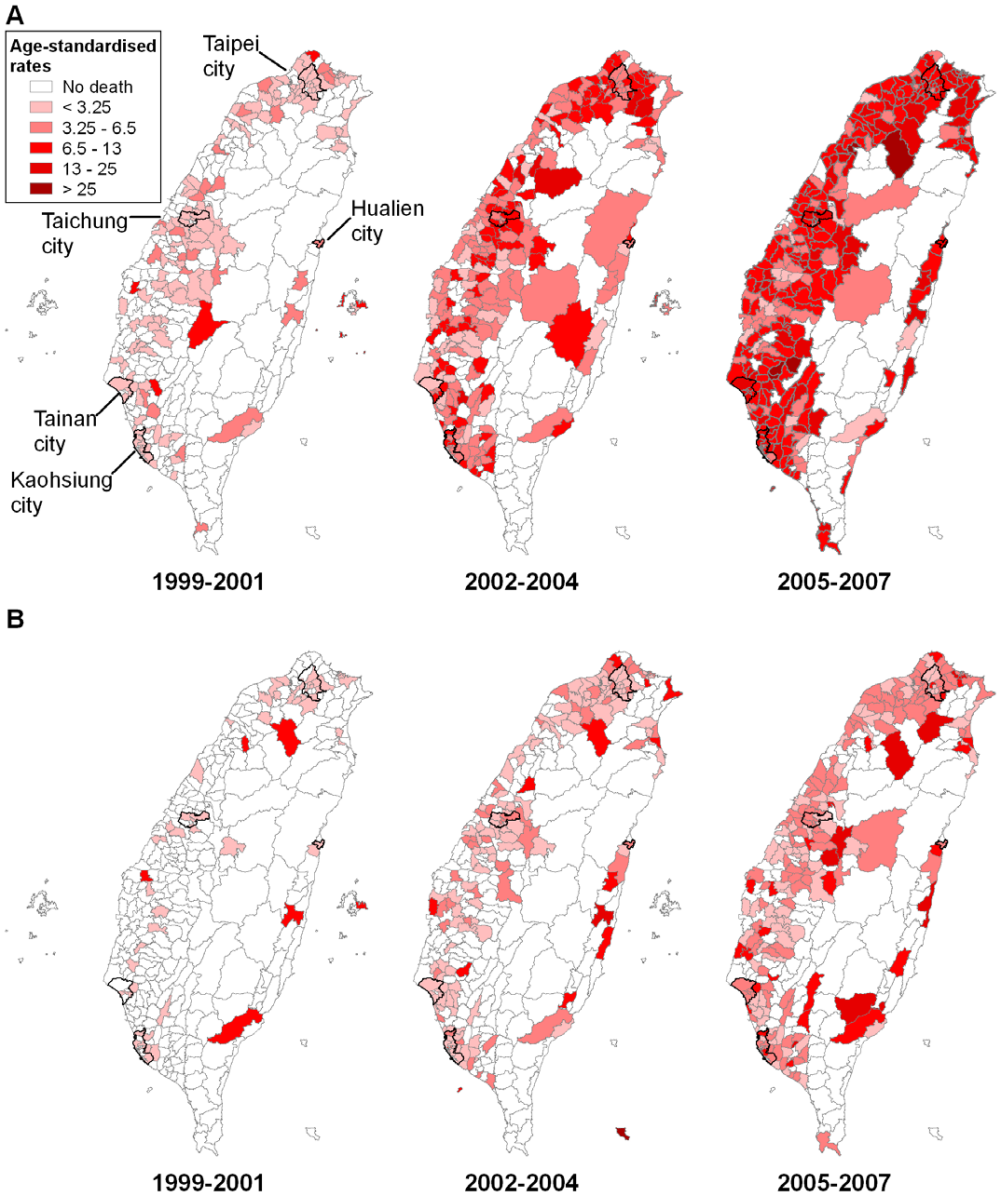
Maps of (unsmoothed) age-standardised rates of suicide (including registered suicide and undetermined death) by charcoal burning across 358 townships in Taiwan, 1999–2001, 2002–2004, and 2005–2007, with five major cities highlighted. (A) Males; (B) females.

Figure 2 shows the maps of raw (unsmoothed) age-standardised rates of charcoal-burning suicide in males and females, for the periods 1999–2001, 2002–2004, and 2005–2007. In the early years of the epidemic (1999–2001), charcoal-burning suicides were mainly seen in West Taiwan, mostly but not entirely in urban areas. There was no evidence for a single geographic point of origin for the epidemic. High rates were also seen in a few rural, mountainous townships, but because their populations were small, a small excess of deaths could lead to extreme estimates of rates. In 2005–2007 charcoal-burning suicides were seen in most areas in Taiwan except some of the eastern, rural townships.

Rates of charcoal-burning suicide were consistently higher in areas with higher levels of urbanisation and higher in men than women throughout the epidemic (Table 1). Overall, there was an urban–rural gradient in both rates and the proportions of charcoal-burning suicide from the early to the most recent years of the epidemic. In women, rates in areas with urbanisation level 5 were low; this category contained the smallest population amongst all levels (2.3% of total population) and thus estimates were unstable.

**Table 1:** Age-standardised rates for all suicides and charcoal-burning suicides and the proportions of charcoal-burning suicide (with 95% confidence intervals) in Taiwan by sex, time period (1999–2001, 2002–2004, 2005–2007), and urbanisation level.

Urbanisation Level	1999–2001			2002–2004			2005–2007		
	Rate		Proportion of CB Suicide	Rate		Proportion of CB Suicide	Rate		Proportion of CB Suicide
	All Suicides	CB Suicides		All Suicides	CB Suicides		All Suicides	CB Suicides	
**Male**									
1 (Most urban)	21.3	1.3	6.1 (4.9–7.7)	27.2	6.6	24.3 (21.8–27.1)	31.5	11.4	36.1 (33.1–39.3)
2	23.1	1.3	5.5 (4.4–6.7)	29.6	6.4	21.7 (19.7–23.9)	32.5	10.9	33.5 (31.1–36.2)
3	26.1	1.4	5.2 (4.1–6.6)	30.8	6.2	20.2 (18.0–22.7)	38.1	11.3	29.8 (27.3–32.5)
4	26.9	1.3	5.0 (3.8–6.5)	31.3	5.7	18.0 (15.8–20.6)	34.4	9.4	27.2 (24.5–30.3)
5	26.6	0.8	3.2 (1.5–6.7)	26.7	2.9	10.7 (6.9–16.6)	36.9	7.9	21.5 (16.0–28.7)
6	32.0	0.9	2.7 (1.5–4.6)	34.8	2.9	8.4 (6.1–11.5)	40.0	8.1	20.2 (16.5–24.7)
7 (Most rural)	31.4	0.7	2.2 (1.1–4.3)	30.4	3.8	12.6 (9.4–16.8)	37.8	7.3	19.3 (15.6–24.0)
**Female**									
1 (Most urban)	11.1	0.2	2.0 (1.1–3.3)	12.8	2.6	20.0 (16.9–23.7)	15.1	4.2	27.5 (24.0–31.4)
2	12.6	0.4	3.2 (2.2–4.5)	14.4	2.5	17.2 (14.8–20.0)	15.8	4.3	27.4 (24.5–30.8)
3	13.0	0.3	2.0 (1.2–3.4)	15.3	2.0	13.2 (10.8–16.2)	15.6	3.9	25.3 (21.9–29.3)
4	12.9	0.2	1.5 (0.7–3.0)	14.3	2.0	14.3 (11.4–18.0)	15.7	3.3	20.7 (17.2–24.8)
5	14.5	0	—	12.9	0.9	6.6 (2.7–16.2)	15.5	0.9	5.8 (2.4–14.4)
6	16.2	0.3	1.7 (0.5–5.3)	17.3	1.2	7.0 (4.0–12.2)	16.2	2.6	15.9 (10.7–23.6)
7 (Most rural)	14.3	0.2	1.1 (0.3–4.4)	15.1	1.6	10.6 (6.5–17.2)	17.3	3.0	17.3 (12.1–24.7)

CB, charcoal-burning.

### Spatial Patterning: Charcoal-Burning Versus Non-Charcoal-Burning Suicides

**Figure 3:**
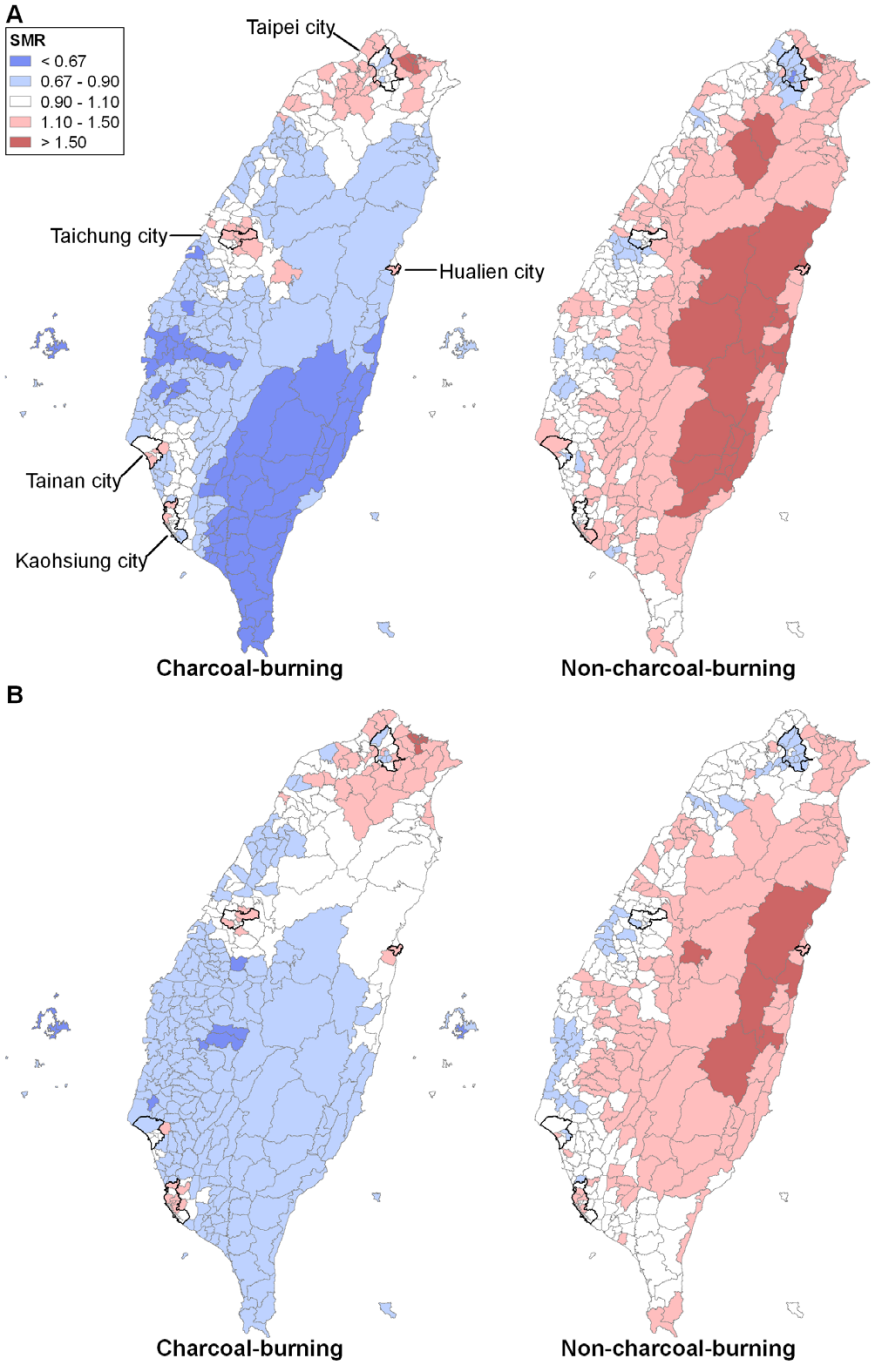
Maps of SMRs for charcoal-burning and non-charcoal-burning suicides (including registered suicides and undetermined deaths) across 358 townships in Taiwan, 1999–2007, with five major cities highlighted. (A) Males; (B) females.

Figure 3 shows the globally and locally smoothed maps of suicides and undetermined deaths by charcoal burning and other methods, for males and females, in the years 1999–2007. There were 4-fold (males) and 10-fold (females) differences in smoothed SMRs for charcoal-burning suicide. Smoothed SMRs ranged from 0.39 to 1.70 (90% range 0.57–1.26) in men and 0.16 to 2.05 (90% range 0.68–1.26) in women. The maps displayed clear evidence of spatial patterning that was similar in men and women; high SMRs for charcoal burning suicides were seen in all three major metropolitan regions of West Taiwan, with the capital Taipei city being somewhat spared, and in the only city of East Taiwan (Hualien). On the basis of the deviance information criterion (DIC), the models accounting for both the global and local variability across areas showed better goodness of fit than models accounting for global variability only, and at least equal fit in comparison to models accounting for local variability only. Therefore we presented the maps of SMRs smoothed both globally and locally here. The models estimated that 89.2% (95% credible interval [CrI] 74.9%–97.4%) of the total area variability in male charcoal-burning suicides could be attributed to the local component, and the corresponding figure for females was 85.0% (95% CrI 59.6%–97.4%), indicating that local characteristics contributed to the majority of the variability of SMRs across townships. Moran's *I* statistics showed evidence of spatial autocorrelation (*I*: 0.31 [*p* = 0.001] for males, 0.11 [*p* = 0.004] for females).

The spatial patterning of charcoal-burning suicide was little changed when data on deaths aged 25–44 y only were analysed (unpublished data). Analyses on data excluding undetermined deaths also showed similar patterns, except that high SMRs in the middle metropolitan area were less evident (unpublished data). When including accidental deaths from pesticide poisoning and suffocation, the spatial patterns of non-charcoal-burning suicides remained generally the same (unpublished data). Spatial patterns of unsmoothed SMRs were broadly similar to those in the smoothed maps, but the patterns were less clearly displayed because there were no charcoal-burning suicides in many townships (35/358 [9.8%] in men, 101/358 [28.2%] in women) (unpublished data).

Smoothed SMRs for non-charcoal-burning suicides showed less between-area variability than those for charcoal-burning suicides: they ranged from 0.53–1.89 in men (90% range 0.83–1.52) and from 0.70–1.75 in women (90% range 0.86–1.38). Non-charcoal-burning suicides had a completely different spatial distribution compared with charcoal-burning suicides (Figure 3). Rural areas in East Taiwan had higher smoothed SMRs than the western, urban areas.

### Secular Trends in Rural–Urban Difference in Suicide

**Figure 4:**
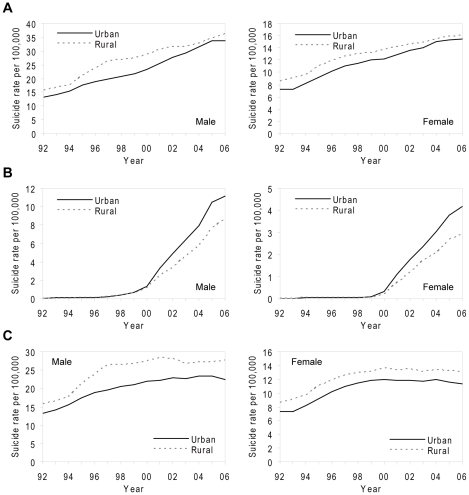
Secular trends in age-standardised rates (3-y moving averages) of suicide (including registered suicide and undetermined death) for males and females in Taiwan, 1991–2007. (A) Overall rates; (B) rates of charcoal-burning suicide; (C) rates of non-charcoal-burning suicide.

Figure 4 shows secular trends in rates of overall, charcoal-burning and non-charcoal-burning suicide and undetermined death (3-y moving averages) for the period 1991–2007 in urban and rural areas of Taiwan, separately for males and females. Overall suicide rates increased throughout the period, but rural–urban differences changed over time (Figure 4A). In the early 1990s overall suicide rates were higher in rural than urban areas; the differences widened in the mid-1990s in men but narrowed markedly after 2000 in both men and women. The rural–urban difference reached its peak in men in the years 1996–1998, when it was 6.7 (95% confidence interval [CI] 5.1–8.4) per 100,000; the corresponding figure in women was 1.6 (0.4–2.8) per 100,000. However, by 2004–2006 the rural–urban differences had almost disappeared (0.9 [−0.9 to 2.8] per 100,000 men and 0.6 [−0.7 to 1.8] per 100,000 women).

The reduction in rural–urban differences in overall suicide rates was largely attributable to the disproportionately greater rise in charcoal-burning suicide in urban than rural areas (Figure 4B), whilst the rural–urban differences in non-charcoal-burning suicide persisted throughout the study period (Figure 4C). Rates of non-charcoal-burning suicide increased during the 1990s but stabilised after 1998, when the epidemic of charcoal-burning suicide emerged. The patterns were little changed when excluding undetermined deaths or including accidents by pesticide poisoning and suffocation (unpublished data).

## Discussion

The recent epidemic of suicide by charcoal burning in Taiwan showed a distinct spatiotemporal pattern in its emergence. The epidemic emerged more prominently in urban areas and there was no evidence for a single geographic area of origin. Rates of charcoal-burning suicide were consistently higher in the metropolitan and city regions than in rural areas. The reduction in rural–urban differences in overall suicide rates since around 2000 appeared to be attributable to the epidemic of charcoal-burning suicide that emerged to a greater extent in urban than rural areas.

### Strengths and Limitations

This study is, to our knowledge, the first national investigation of the geographic patterning of a major epidemic of suicide. The availability of township-level data allowed a detailed investigation of the geographic distribution of the epidemic across small areas. We used Bayesian hierarchical models to estimate smoothed SMRs allowing for both global and local variability, and hence to tackle the problem that estimates of SMRs are imprecise in areas with small populations. However our study has a number of limitations. Not all suicides in Taiwan with ICD-9 codes E952/E982 are charcoal-burning suicides: a proportion will be from poisoning using other sources of nondomestic gas such as car exhaust fumes. However, data for 2002 indicate that only 10% of E952/E982 cases died from car exhaust gassing [14], although this proportion may have changed over time and thus affected the estimated contribution of charcoal-burning suicide to suicide rates by nondomestic gas poisoning. Not all undetermined deaths will be suicides [13], but the sensitivity analyses on recorded suicides alone gave similar results. The measurement of urbanisation is complex. Our indicator is the one used by the Taiwanese government and is a synthesis of previously agreed measures [15].

### Possible Explanations for the Spatial Patterning of Charcoal-Burning Suicide

The epidemic of charcoal-burning suicide in Taiwan appeared to begin in mainly West Taiwan. The lack of a single area of origin is compatible with a diffuse geographic effect of media publicity and imitation [9]. Furthermore, throughout the epidemic high rates appeared to cluster in major metropolitan and city regions. This clustering indicates that the effect of media was greater in urban areas or that the method was more easily available/easier to implement in these areas. The urban environment (e.g., higher levels of media exposure and easy access to barbecue charcoals [1]) or certain characteristics of the vulnerable population living in cities (e.g., rapid acquisition of knowledge and accepting attitude towards a new suicide method [3]) may have contributed to the observed geographic distribution.

The stories of charcoal-burning suicide in television news, newspapers, and Internet articles may have triggered imitative suicides that occurred across geographic boundaries [3]. It has been suggested that the sensational coverage of charcoal-burning suicides and the detailed portrayal of the method by media may have contributed to the epidemic in Hong Kong [1],[5]. A study of suicide attempters using charcoal burning in Hong Kong showed that nearly all survivors learnt of the method from the newspaper [5]. The first charcoal-burning suicide victim in Taiwan reportedly gained knowledge of the method from a Hong Kong newspaper website [28]. Although there has been no study investigating the impact of the media's reporting style on the development of the epidemic of charcoal-burning suicide in Taiwan, a recent study shows that Taiwanese media coverage of a celebrity's suicide in 2008 was sensational and included details of the method used (charcoal burning) [29]. Such reporting is believed to contribute to the risk of imitative suicides; indeed the study also found that suicide attempters using the same method as the celebrity's suicide (charcoal burning) were more likely to report being influenced by the media coverage than those using other methods [29].

The concentration of charcoal-burning suicides in the major metropolitan and city areas of Taiwan suggests that the impact of media may be greatest in these urban regions. The finding of higher rates of charcoal-burning suicide in urban areas was not an artefact of higher underlying suicide rates, because the proportions of charcoal-burning suicide consistently showed an urban–rural gradient (Table 1). This gradient indicates that vulnerable populations in urban areas may have adopted the new suicide method more readily than their rural counterparts. Residents living in urban regions may be exposed to the television or newspaper reporting more frequently than those living in rural areas. For example, in the capital Taipei city, 92% and 66% of households were regular consumers of cable TV and newspapers, respectively, in 2000; the corresponding figures for Nantou, a rural county of central Taiwan, were 63% and 37% [30]. Urban areas also had easier access to Internet than rural areas: in 2003, a government report showed that 73% of the households were connected to Internet cable in Taipei city, compared with 25% in Nantou county [31].

In Taiwan, barbecue charcoals are mostly sold in supermarkets or convenience stores, which are mainly located in urban or suburban areas. Therefore at-risk urban residents may have easier access to the method than those living in rural areas when charcoal burning is the choice of suicide method. In Hong Kong, 80% of the charcoal-burning suicides occurred in urban areas [1]. The urban overrepresentation of charcoal-burning suicides is thought to be associated with easy access to barbecue charcoals, which are readily available in supermarkets [1]. Past research in Taiwan indicates that accessibility to lethal methods is associated with method-specific suicide rates. At county level, rates of suicide by solids/liquids poisoning (most of these suicide incidents involved pesticides) were correlated with the proportion of agricultural population, an indicator of accessibility to pesticide, and suicide rates by jumping were correlated with the proportion of households living in high buildings, an indicator of accessibility to high places to jump from [32]. Area differences in method-specific suicide rates were also seen across different countries in the world [33] and across smaller areas within one country [34] or region [35]. The differences are thought to be mainly due to different accessibility to lethal methods of suicide [33]–[35].

### The Impact of Charcoal-Burning Suicide on Trends in Rural–Urban Differences

The rural excess in suicide risk prior to the appearance of charcoal-burning suicides was greatly reduced when the epidemic emerged more prominently in urban than rural areas of Taiwan. This trend was in contrast to what occurred in several western countries in recent decades. In the West, rises in suicide rates, particularly for males, were greater in rural than urban areas [36]–[39], and the rural–urban differences appeared to widen in some countries [36],[39]–[41]. In England and Wales, unfavourable trends in suicide for young people generally occurred in areas remote from main populated centres and the patterns could not be explained by changes in the lethality of popular suicide methods [38]. In contrast, our results showed that the area difference in rates of suicide by charcoal burning in Taiwan had a large impact on the rural–urban difference in overall suicide rates.

### Implications for Suicide Prevention

Taiwan has experienced an upward trend in suicide rates since the early 1990s. Rises in unemployment may have contributed to these increases, although unemployment rates declined after reaching a peak around 2002 [42],[43]. The emergence of charcoal-burning suicides contributed to recent increases in suicide rates in Taiwan. In both Hong Kong and Taiwan, the emergence of charcoal-burning suicides was not accompanied by corresponding increases in suicides by other methods [3],[27]. Furthermore, charcoal-burning suicides showed different sex and age patterns from those of suicides by other methods [13],[27] and had a distinct geographic distribution, as demonstrated in our study. These observations suggest that many victims of charcoal-burning suicide would not otherwise have died by suicide if this novel method were not available. Prevention measures targeted at the method may help reverse the recent rises in suicide rates in Taiwan.

The geographic pattern of the epidemic of charcoal-burning suicide suggests that media coverage and easy assess to barbecue charcoal may have contributed to the epidemic. A better understanding of the causes of this epidemic will inform suicide prevention in Taiwan, particularly in urban areas where risk is concentrated, and has implications for other countries wishing to avoid such a public health disaster. Responsible media reporting of suicide stories, following guidelines that are now readily available [44],[45], may help prevent the spread of a new suicide method. Restricting the access to barbecue charcoals, particularly in urban areas of Taiwan, with measures such as placing charcoal bags behind supermarket counters or banning the sale in convenience stores may also help offset the unfavourable trends [1],[5]. Responsible media reporting is essential to prevent the spread of a new suicide method with high lethality such as charcoal burning in regions where this suicide method is not yet widely known [1]. However, in countries like Taiwan where the epidemic of charcoal-burning suicide is already established, the controls of environmental factors such as access to barbecue charcoal may be more effective since it takes time for the perception of this particular method of suicide to be changed by responsible media reporting and public education [1]. The distinct spatiotemporal pattern of charcoal-burning suicide in Taiwan shown here has potential public health implications for comprehensive suicide prevention strategies.

## Supporting Information

Alternative Language Abstract S1 Chinese translation of the abstract by SSC (traditional Chinese characters).
(0.03 MB DOC)

Alternative Language Abstract S2 Chinese translation of the abstract by SSC (simplified Chinese characters).
(0.03 MB DOC)

## Abbreviations

SMR: standardised mortality ratio
