# Supplementary material for: The Evolution of the Epidemic of Charcoal-Burning Suicide in Taiwan: A Spatial and Temporal Analysis
Source: PLoS Med. 2010 Jan 5;7(1):e1000212. doi: 10.1371/journal.pmed.1000212 (PMC2794367; doi:10.1371/journal.pmed.1000212)
Supplement: Alternative Language Abstract S1 — Chinese translation of the abstract by SSC (traditional Chinese characters). (0.03 MB DOC) [file pmed.1000212.s001.doc]

# Translation of the abstract into complex Chinese by Shu-Sen Chang

# 摘要（Abstract）

**背景（Background）**

在過去十年間，東亞發生了一波以燒炭造成一氧化碳中毒而自殺身亡的流行現象。我們研究了此波流行的地理變異和趨勢變化，以分析其對台灣自殺之流行病學的影響。

**研究方法與發現（Methods and Findings）**

針對因燒炭中毒身亡而被歸類為自殺或不明死因的個案，分別對1999-2001年、2002-2004年與2005-2007年三個時期，我們計算出台灣各鄉鎮市區（15歲以上人口的中位數為27,000人）的燒炭自殺率。利用貝式階層統計模型，我們計算出各鄉鎮市區燒炭與燒炭以外自殺之標準化死亡比的平滑估計值。針對1991-2007年間，我們比較了城市與鄉村地區整體與燒炭自殺率的趨勢變化。結果顯示，燒炭自殺的流行現象，在城市地區比鄉村地區顯著，並且流行的發生沒有一個特定的地理起始點。整體而言，大都會地區是燒炭自殺率最高的區域。在1998年之前，鄉村的整體自殺率高於城市，但因燒炭自殺率的增加是城市高於鄉村，近年來台灣整體自殺率的城鄉差異已消失。

**結論（Conclusions）**

燒炭自殺的流行，對於台灣自殺的地理分布有顯著影響。過去十年間台灣燒炭自殺的地理分布與趨勢變化模式，可能與媒體廣泛報導此自殺方式和烤肉炭的便於取得這兩個因素有關。如能針對這兩個因素予以介入，例如媒體能夠依循自殺報導的準則，以及限制烤肉炭的可近性，可能有助於抑止燒炭自殺的增加。
