# Supplementary material for: The Evolution of the Epidemic of Charcoal-Burning Suicide in Taiwan: A Spatial and Temporal Analysis
Source: PLoS Med. 2010 Jan 5;7(1):e1000212. doi: 10.1371/journal.pmed.1000212 (PMC2794367; doi:10.1371/journal.pmed.1000212)
Supplement: Alternative Language Abstract S2 — Chinese translation of the abstract by SSC (simplified Chinese characters). (0.03 MB DOC) [file pmed.1000212.s002.doc]

# Translation of the abstract into simplified Chinese by Shu-Sen Chang

# 摘要（Abstract）

**背景（Background）**

在过去十年间，东亚发生了一波以烧炭造成一氧化碳中毒而自杀身亡的流行现象。我们研究了此波流行的地理变异和趋势变化，以分析其对台湾自杀之流行病学的影响。

**研究方法与发现（Methods and Findings）**

针对因烧炭中毒身亡而被归类为自杀或不明死因的个案，分别对1999-2001年丶2002-2004年与2005-2007年三个时期，我们计算出台湾各乡镇市区（15岁以上人口的中位数为27,000人）的烧炭自杀率。利用贝式阶层统计模型，我们计算出各乡镇市区烧炭与烧炭以外自杀之标准化死亡比的平滑估计值。针对1991-2007年间，我们比较了城市与乡村地区整体与烧炭自杀率的趋势变化。结果显示，烧炭自杀的流行现象，在城市地区比乡村地区显着，并且流行的发生没有一个特定的地理起始点。整体而言，大都会地区是烧炭自杀率最高的区域。在1998年之前，乡村的整体自杀率高於城市，但因烧炭自杀率的增加是城市高於乡村，近年来台湾整体自杀率的城乡差异已消失。

**结论（Conclusions）**

烧炭自杀的流行，对於台湾自杀的地理分布有显着影响。过去十年间台湾烧炭自杀的地理分布与趋势变化模式，可能与媒体广泛报导此自杀方式和烤肉炭的便於取得这两个因素有关。如能针对这两个因素予以介入，例如媒体能够依循自杀报导的准则，以及限制烤肉炭的可近性，可能有助於抑止烧炭自杀的增加。
